# Cannabidiol attenuates precuneus activation during appetitive cue exposure in individuals with alcohol use disorder

**DOI:** 10.1007/s00406-025-01983-4

**Published:** 2025-03-18

**Authors:** Tristan Hurzeler, Warren Logge, Joshua Watt, I. S. McGregor, Anastasia Suraev, Paul S. Haber, Kirsten C. Morley

**Affiliations:** 1 Specialty of Addiction Medicine, Sydney Medical School, Faculty of Medicine and Health, University of Sydney, Sydney, NSW Australia; 2 Edith Collins Centre for Translational Research (Alcohol, Drugs & Toxicology), Royal Prince Alfred Hospital, Sydney Local Health District, Sydney, NSW Australia; 3 Lambert Initiative for Cannabinoid Therapeutics, University of Sydney, Sydney, NSW Australia; 4 Faculty of Science, School of Psychology, University of Sydney, Sydney, NSW Australia

**Keywords:** Cannabidiol, Neuroimaging, Cue reactivity, Alcohol use disorder, Pharmacotherapy

## Abstract

Alcohol use disorder (AUD) is a prevalent psychiatric condition characterised by problematic alcohol consumption and craving, necessitating the exploration of novel therapeutic interventions. Cannabidiol (CBD), a non-psychoactive component of cannabis, has shown potential in modulating neural processes associated with substance use disorders including AUD. This study aimed to investigate the effect of CBD on alcohol cue-induced activation of neurocircuitry associated with alcohol craving, and impact on mood, craving, and cognitive functioning in individuals with AUD. In a cross-over, double-blind, randomized trial, 22 non-treatment seeking individuals (M = 29 years) diagnosed with AUD (DSM-V) received either 800 mg of CBD or a matched placebo, completing two respective fMRI sessions. The primary outcome was neural activation in response to alcohol versus control visual cues, measured using a functional magnetic resonance imaging (fMRI) alcohol cue reactivity task. Secondary outcomes included assessments of mood, craving, and cognitive functioning. Region of interest analyses showed no differences in alcohol cue-elicited activation between the CBD and placebo conditions. However, exploratory whole-brain analysis indicated a significant treatment effect of CBD in the precuneus which was independent of cue specificity. There were no significant treatment effects of CBD compared to placebo on acute craving, mood, or cognitive functioning. In non treatment seeking individuals with AUD, CBD modulates precuneus activity during alcohol cue exposure. Further studies examining the effect of CBD on treatment-seeking AUD individuals are warranted.

**Supplementary Information:**

The online version contains supplementary material available at 10.1007/s00406-025-01983-4.

## Introduction

Alcohol use disorder (AUD) is characterised by compulsive, chronic alcohol use despite adverse social, occupational, or health consequences [1]. Heavy, chronic alcohol use perturbs functioning within regions of the meso-corticolimbic [2, 3], salience networks [4], limbic networks [5] as well as fronto-striatal functional connectivity [6] which are thought to underpin the compulsive seeking behaviour and cognitive processes which maintain AUD [7]. Pharmacotherapies targeting these dysregulated neurobiological systems aim to manage the core symptoms of AUD and improve clinical outcomes by facilitating behavioural change. However, TGA approved medication for the management of AUD (naltrexone, disulfiram (Antabuse), and acamprosate [8–10]), are only modestly effective and there is a large degree of heterogeneity in treatment response [11] wherein new pharmacotherapeutic options are required [12].

Cannabidiol (CBD), a non-intoxicating component of *Cannabis sativa*, has emerged in recent years as a potential treatment target for AUD [13]. CBD is a non-intoxicating phytocannabinoid with known anti-seizure properties [14] and a favourable side effect profile[15–17] . It is a CB1R non-competitive allosteric modulator [18], mediating anandamide (AEA) transportation by targeting fatty acid-binding proteins (FABP). CBD inhibits FABP catabolism of AEA and reduces cellular uptake of endocannabinoids [19] and modulates dopaminergic release [20] in regions of networks involved in cue induced craving and regulatory behaviours [21] including mesocorticolimbic, salience, and fronto-striatal networks in healthy brains. CBD may therefore normalise functions characteristic of AUD such as reward anticipation, emotion regulation, salience processing, and executive functioning.

CBD has shown promise for the management of cue-induced craving in both preclinical models and clinical samples with substance use disorders. Preclinical sutides have demonstrate that acute doses of CBD have been shown to reduce stress and alcohol cue reinstatement in preclinical models [22, 23]. The only previous clinical study of CBD in a substance use disorder to-date demonstrated that CBD, compared to placebo, reduced opiate cue-induced craving and anxiety for those with opioid use disorder [24]. Further, CBD induced decreases in attentional bias to cigarettes for those with nicotine dependence [25] suggests a capacity to normalise the appraisal of drug associated cues as hyper-salient. While THC disrupts salience network activation, neuroimaging studies have found that CBD restores salience network functionality [26]. CBD also normalises insular dysfunction during motivational salience processing for those with clinical high risk of psychosis [27]. CBD may thus have potential to manage cue induced craving in AUD through the regulation of hyper-sensitivity to alcohol associated cues, however, the effect of CBD on alcohol cue reactivity and neurocircuitry in the AUD population has not been studied to-date.

Cue-reactivity neuroimaging paradigms are a well-validated paradigm for investigating craving associated neuronal activity [28] and provide a method for examining the mechanism of action of alcohol treatments and to identify preliminary signals of efficacy. Previous research demonstrates that the dorsolateral PFC, left and right caudate, and bilateral ventromedial PFC, among others, have been shown to be relevant in alcohol cue studies in which participants are exposed to alcohol cues [29, 30]. Using a similar fMRI cue reactivity paradigm, we have previously shown that, compared to high dose baclofen, placebo-treated participants demonstrated hyperactivation in the bilateral caudate nucleus and dorsal anterior cingulate cortex and that this activation was positively correlated with heavy drinking days (HDD) [31]. Similarly, naltrexone has been shown to attenuate fronto-striatal regions associated with alcohol-cue induced craving [32] and that the degree of this modulation predicts time to relapse [33].

As previously outliend [34], we aimed to conduct a double-blind, within-subject cross-over, randomised trial in individuals with AUD to determine the effect of CBD versus placebo on blood-oxygen-level-dependent functional magenetic resonance imaging (BOLD fMRI) and self-reported alcohol craving, mood and cognitive functioning. We hypothesised that mesocorticolimbic brain activation (e.g., dorsolateral PFC, left and right caudate, and bilateral ventromedial PFC), as measured using the blood oxygen level dependent (BOLD) signal when exposed to an alcohol cue, will be significantly attenuated in CBD-treated AUD individuals compared to those on placebo.

## Materials and method

### Design

The trial was conducted over a 48-month period at the Royal Prince Alfred Hospital (RPAH) in Sydney, NSW Australia between 2021 and 2023. The study was approved by the Human Ethics Review Committee of the Sydney Local Health District (X19-0416) and sponsored by the Sydney Local Health District. The trial was registered in the Clinical Trials Registry (NCT05387148) and the protocol (including design and analytic plan) was previously published [34]. Twenty-two non-treatment seeking individuals who met the Diagnostic and Statistical Manual of Mental Disorders, 5th edition (DSM-V) [35], criteria for current AUD were recruited to participate in a randomised, double-blinded, placebo-controlled, crossover trial including 3 days of data collection. Here, we present results from the cue reactivity fMRI paradigm (day 2). Recruitment streams consisted of clinical referral from treating physicians, nurses, and psychologists among RPAH outpatients as well as via flyers/community advertisements at local general practitioners, newspapers, and websites. All participants provided written informed consent prior to randomisation and the commencement of the trial.

### Participants

Twenty-two participants were recruited following screening for inclusion and exclusion criteria. *Inclusion criteria*: a) Male and female patients between the ages of 18 and 65 meeting DSM-V criteria for current AUD; b) Adequate cognition and English language skills to be able and willing to give valid informed consent and complete research interviews; c) Must have a stable housing and be able to nominate a reliable contact person for locating them if necessary.

*Exclusion criteria*: a) Active major psychological disorder associated with psychosis or significant suicide risk; b) Pregnancy or lactation. Women were advised to use reliable contraception throughout the duration of drug therapy, and a urine pregnancy test was conducted as necessary; c) Dependence on any substance other than nicotine (e.g., methadone); d) Diagnosis of epilepsy and/or current use of anti-epileptic drugs; e) Liver failure with jaundice or prolonged INR above 1.3; f) Medical complications such as liver failure, cardiac ischemia or conduction abnormalities, renal impairment or unstable elevated vital signs (systolic blood pressure > 180, diastolic blood pressure > 120, or heart rate > 150); g) Severe cognitive impairment or insufficient English or literacy to complete study processes; h) Concurrent use of drugs potentially exacerbated by CBD via CYP3A5, including cardiac medication (eg betablockers, calcium channel blockers, and statins), macrolides and recent antihistamine use; i) Claustrophobia; j) Extreme obesity; k) Previous brain surgery; l) Unable to complete an MRI scan (e.g., metal implants, previous emoployment as a machinist, welder or metal worker).

### Procedure

After meeting the eligibility criteria through structured interview and medical evaluations, participants provide informed consent. They were then randomly assigned to one of two treatment regimens: either receiving 800 mg CBD (4 × 200 mg gel capsules per day, days 1–3) or matched placebo for two consecutive days. Following an average washout of 29.2 days (SD = 11.25), participants received the alternate treatment allocation at the subsequent session.

On the first testing day (T1), participants presented to the RPAH to receive the first dose of CBD or Placebo and complete a series of questionnaires as detailed above. On the second day (T2), participants received their second dose under supervision then completed a battery of executive functioning tasks, followed by assessments of craving and mood (see below). They were then escorted to an imaging facility for an MRI scan conducted 90 min after their second dose of CBD/matched placebo, aligned with literature suggesting C_max_ occurs between 1 and 4 h post-dose [16, 36, 37]. Prior to imaging, participants underwent a blood alcohol content (BAC) reading (BAC = 0.00 was required), followed by a structural scan and functional cue reactivity task-based acquisition. Craving was assessed after alcohol and control cue blocks, as well as before and after the scan. Following washout, participants subsequently received the alternate capsules and completed the same testing procedure. Following completion of the repeated testing days, participants underwent a follow-up over the phone, with an average of 34 days (SD = 15.7) between final testing day and follow-up.

### Interventions

Softgel capsules containing 200 mg CBD in medium chain triglyceride (MCT) oil (manufactured by Linnea SA, Lavertezzo, Switzerland) [38] and matching placebo (softgel capsules containing only MCT oil) was purchased from BOD Science [39]. The matching placebo was identical in appearance, taste, and composition except for the active ingredient of pure CBD. A three-day consecutive dose regimen was administered as follows: T1 (Day 1): 4 × 200 mg followed by T2 (Day 2 Experimental Session 1): 4 × 200 mg and then T3 (Day 3 Experimental Session 2): 4 × 200 mg. Participants were administered CBD and placebo orally with water, under supervision, to ensure compliance with the dose regimen.

### Randomisation and allocation concealment

This study was conducted under double-blind conditions such that participants and study staff were unaware of medication assignment. A computer-generated random allocation procedure was conducted through REDCap (Research Electronic Data Capture; a secure web application for building and managing online surveys and databases; [40]) by the allocated RPAH pharmacist.

### Measures

#### Sample characteristics

The following questionnaires were presented on Day 1, and responses collected online using REDCap: (1) Demographics, medical history, personal and family history of AUD, and alcohol treatment history as collected in previous research [41]; (2) a structured psychiatric diagnostic interview using the Mini-International Neuropsychiatric Interview (MINI) [42] (3) recent (last 28 days) alcohol consumption (frequency/quantity) assessed by the Timeline Followback Method (TLFB; [43]); (4) severity of alcohol dependence assessed by the Alcohol Dependence Scale (ADS; [44]); (5) craving for alcohol measured by the Penn Alcohol Craving Scale (PACS; [45]; (6) symptoms of depression, anxiety and stress as measured by the Depression Anxiety Symptom Scale (DASS) (Lovibond & Lovibond, 1995); (7) sleep problems as assessed by the Insomnia Severity Index (ISI; Bastien, Vallieres, & Morin, 2001).

#### Craving and mood

The following self-reported questionnaires were collected on Day 2 using REDCap unless preformed within the scanner for which Inquisit 4 [46] was used: (1) Positive and Negative Affect Schedule (PANAS; [47]), with higher scores indicating higher positive and negative mood states pre- and post- scan; (2) Alcohol Urge Questionnaire (AUQ) was used to assess expectancy of alcohol effects and urge to drink alcohol [48]; (3) 11-point Visual Analogue Scale (VAS) was used to assess alcohol craving, thirst, and anxiety, and was captured before, during, and afte the scan.

#### Executive functioning tasks

A 30-min neurocognitive battery of tasks measuring executive function was administered via Inquisit 4 before the scan on Day 2. These tasks included: (1) The Balloon Analogue Risk Task (BART; [49]). Participants are instructed to pump a balloon to earn as many points as possible, risking a pop that erases points for that balloon. Participants must complete 50 trials, with trial end signified either by a balloon pop (unsuccessful) or collection of trial earnings (successful). The overall number of pumps for successful trials assess self-regulation and adaptive risk taking; (2) Columbia Card Task, Hot Version with delayed feedback (CCT; [50]): a task where 32 cards are presented facedown, participants are instructed to select as many cards they would like for points before requesting them to be revealed. All selected cards are then shown to the participants one-by-one with points being awarded for every “win” card until there is none left, or a “loss” card is encountered, whereby points are subtracted from the total gained thus far in the trial. The average number of cards turned over in the task is a score of inhibition, working memory updating, task-set switching, and attention.

Participants were also administered a paper version of the Trail Making Task (TMT), comprising of Part A and B (TMT-A and TMT-B) [51]. In TMT-A, participants must connect consecutively numbered circles as quickly as possible, while in TMT-B, they alternate between numbers and letters (1-A-2-B-3-C, etc.). Difference scores were derived by subtracting completion times for TMT-A from TMT-B, with shorter completion times indicating better executive functioning.

#### Cue reactivity task

An adapted cue reactivity task [52] was presented to measure alcohol cue-elicited brain activity. Stimuli comprised of two types: alcohol-related pictures depicting types of alcohol (lager/wine/spirits) and drinking situations; and a control type comprising of validated, neutral pictures matched for colour and complexity. Images were presented on an MRI-compatible screen for 6.6 s in blocks of three images of the same type. Ten alcohol and six neutral blocks presented throughout the experiment with stimuli and the block order was randomised [16 total blocks, 646 s]. Following each block, an 11-point VAS was presented and participants responded using an MRI-compatible two-button response pad (Cedrus Corporation; San Pedro) within a 10 s window (see supplementary files for task procedure).

### MRI acquisition

Participants were scanned using a 3-Tesla GE Discovery scanner and a 32-channel head coil. A structural scan (T1 weighted 1-mm^3^ voxel resolution) was acquired for use in pre-processing purposes (TR: 7200 ms, TE 2.7 ms, 176 sagittal slices, 1 mm thick, no gap, 256 × 256 × 256 matrix). For functional imaging, a GE Multiecho “HyperMEPI” echoplanar imaging sequence [53] that employs multiband [3 echo times] fMRI was used, enabling improved signal to inferior regions (e.g., VMPFC etc.) which generally suffer from signal loss [54]. Multiphase volumes of whole brain, comprising 45 axial slices were collected in an ascending interleaved fashion angled 15 degree from the AC-PC line superior to inferior using a Gradient Echo pulse sequence with 3 echoes (TR: 2000 ms, TE_1_ /TE_2_ /TE_3_ = 10/25/40 ms, Flip Angle:70°, FOV:220 mm, matrix of 64 × 64, slice thickness 3 mm; slice gap 0.4 mm; with a voxel resolution 3.44 × 3.44 × 3.44 mm3). Additionally, A B_0_ map was acquired (TR = 1000 ms, TE = 4.6 ms, flip angle 30°, FOV: 220 mm, matrix 64 × 64 and bandwidth 62.5). Task acquisition was 646 s. 323 echoplanar image volumes comprising 45 axial slices were acquired from ventral to dorsal (963 images from three diff TEs). Participants’ heads were fixed with foam pads and a set of MRI compatible headphones to minimise head movement and framewise displacement.

### Image processing

Cue reactivity relevant functional scans were processed using FMRIPrep ([55, 56], RRID:SCR_016216); a summary is provided here, with full preprocessing pipeline presented in Supplementary Material (FMRIprep boiler plate in supplementary). Intensity non-uniformity correction was applied to T1-w images which were then skull-stripped. These T1-w images were then used in brain surface reconstruction with volume-bases structural images segmented and normalised into MNI space.

For each participant, and session a reference volume was generated from the shortest echo of the BOLD run and skull-stripped. Head motion was estimated with respect to the BOLD reference. Respective field maps (B0-nonuniformity map) were co-registered to the EPI reference run and then converted to a displacements field map. A corrected EPI reference was calculated using the estimated susceptibility distortion for a more accurate co-registration with the T1-w image. This displacement field map was then used to better co-register the BOLD reference scan to the T1w. BOLD reference volumes were then co-registered to the T1w reference and the timeseries were resampled onto their original, native space by applying a single, composite transform to correct for head-motion and susceptibility distortions resulting in a pre-processed BOLD timeseries (PP BOLD). fMRIPrep was then leveraged to optimally combine each of the three different echo image sets. To do this the monoexponential signal decay model was fitted to a nonlinear regression to create a T2* map (using T2*/S0 estimates from a log-linear regression fit as initial values). Using the method described in [57], this T2* map was then used to combine PP BOLD across echoes. The remaining were optimally combined PP BOLD time series. These were then distortion-corrected using the B0 field maps, motion correct, co-registered to the T1-w structural data, normalised to MNI space, projected to cortical surface and then resampled to FreeSurfer’s (FreeSurfer 6.0.1, surfer.nmr.mgh.harvard.edu) fsaverage space. fMRI images were then resampled in SPM12 with spatial smoothing using a kernal of 8mm for subsequent fMRI subject modelling. See Supplementary Material for further details (image data preprocessing).

### Statistical analysis

Mean, standard deviation, and proportions were calculated for all relevant sample characteristics using R software (Version 4.0.3). Further relevant state change covariates (previous day drinking, session order, and crossover contrasts defined using crosscarry package [58]) were extracted for use as covariates in subsequent generalised estimation equations (GEE). For all other non-imaging measures including mood, craving and cognitive tasks, GEEs in R software (Version 4.0.3) were used to test for statistically significant differences between treatment (CBD vs placebo) and across time (pre/post scan and alcohol vs control blocks). Interactions were examined for treatment effects across time (ie treatment effects for comparisons between alcohol/control cue exposure blocks or pre/post scan). Implementation of Generalised Estimating Equations (GEE), in addition to the use of the CrossCarry package, accounts for the complexities typical of crossover designs. CrossCarry allows for the incorporation of flexible covariate structures that can explicitly model distinct effects such as carry over effects, order effects, and habituation. In addition, GEE explicitly accounts for within-subject correlation by using a working correlation structure (in this case exchangeable), ensuring accurate estimation of the treatment effects and standard error. Therefore, by leveraging CrossCarry along with GEE our analysis presents a statistically rigorous method for isolating treatment effects while accounting for typical effects such as habituation.

Data analysis for the fMRI cue reactivity task was conducted using SPM12 with two levels. Within the first level (session-specific) analysis two conditions were modelled: alcohol cues and control cues (matched for novelty and visual complexity of the alcohol stimuli). These conditions were modelled as a box-car function convolved with the canonical haemodynamic response. Additionally, six motion correction parameters and VAS blocks were included as regressors of no interest while fixation crosses were considered an implicit baseline.

A priori regions of interest (ROIs) were identified for analysis of activity associated with alcohol cue reactivity. These ROIs comprised the dorsolateral PFC, left and right caudate, and bilateral ventromedial PFC which are highlighted during alcohol cue reactivity in studies examining AUD [59, 60] including treatment effects [30]. The dorsolateral and bilateral ventromedial PFC ROIs were be defined using probabilistic maps extracted from Brainmap database [61, 62], binarized with a threshold of ≥ 0.90. While the Harvard–Oxford subcortical probability atlas (http://www.cma.mgh.harvard.edu/fsl_atlas.html) was used here to define the caudate (caudate body) ROIs. ROIs were extracted using the MarsBar toolbox [63] to obtain unweighted beta estimates within ROIs specific to each task. Relevant betas were collected for both sessions of each participant for each of these regions. As beta weights from Alcohol cue > Control cue contrasts have previously been demonstrated to have low reliability as a contrast when scans repeated [64], alcohol cue exposure blocks compared to implicit baseline (ALC) and control cue exposure blocks compared to implicit baseline (CON) were applied as contrasts at the second level. Generalised estimating equations (GEEs) were conducted in R software (Version 4.0.3). These equations consisted of beta estimates of both contrasts, including a within-subjects factor of treatment (placebo, CBD) and relevant covariates were included to ensure adequate balancing of potential confounding variables (drinking on the previous day, session order and dummy variables to account for cross over effects). By employing the Simple Interactive Statistical Analysis Bonferroni tool (http:// www.quantitativeskills.com/sisa/calculations/bonfer.htm) to balance type I and type II error associated with multiple comparisons correlation between ROIs, an adjusted bonferoni threshold was set at p < 0.03. For task-based results we report beta mean correlation coefficients.

Post hoc whole brain analysis was then conducted to further investigate difference in BOLD signal between post placebo and CBD dosing imaging session using the Multivariate and Repeated Measures for Neuroimaging (MRM) toolbox (version 1.0). MRM is particularly suited to mixed effects statistical modelling of repeated measures designs. First-level ALC and CON contrasts were implemented using GLM at the second-level with 5000 permutations to account for between subject variances*.* Family-wise error (p < 0.05) at the cluster was corrected for by using a cluster-forming height threshold of p = 0.001 as recommended by [65]*.* x, y, z MNI coordinates are reported and the SPM Wake Forest University (WFU) Pickatlas toolbox (http://www.fmri.wfubmc.edu/cms/software, version 3.0.5) used to identify significant cluster regions*.*

## Results

**Fig. 1:**
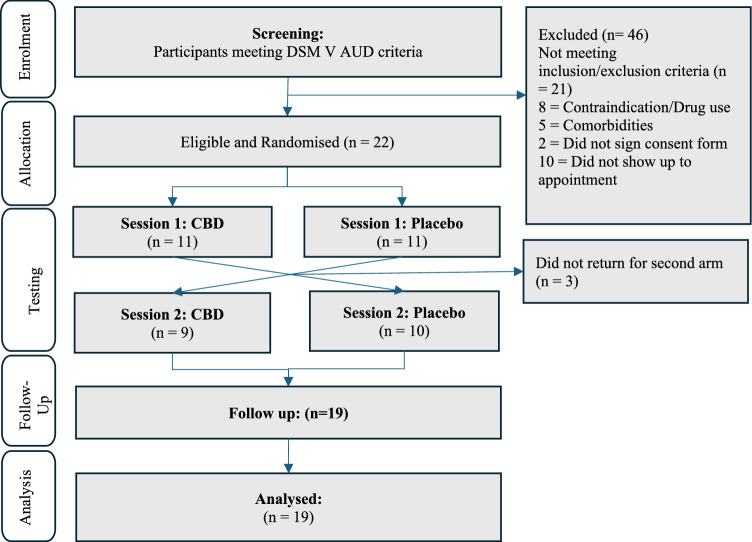
Consort Flow Diagram. Details the flow of participants from recruitment to follow up and includes the information about dropout

Nineteen participants completed both study sessions of the fMRI study (see Fig. 1: Consort flow diagram) with 18 participants yielding complete and usable neuroimaging data.

### Sample characteristics

Baseline sample characteristics are depicted in Table 1. Overall, the mean age of the remaining participants was 29.39 years (SD = 12.83) and participants had received on average 13.95 years (SD = 2.41) of education. Participants, on average, drank 8.41 (SD = 6.02) standard drinks per drinking day, had an ADS score of 15.44 (SD = 7.81) with approximately 8.6 (SD = 9.97) years of problem drinking. Throughout the study, a total of eight side effects were recorded. Among these, four participants experienced side effects during CBD administration while the remaining four reported these following placebo administration. During CBD administration days one participant reported drowsiness, one reported somnolence, and two reported lethargy.

**Table 1:** Sample characteristics

Participants (n = 18)	
Demographics	
Age (years)	29.39 ± 12.83 [18–62]
Education (years)	13.94 ± 2.41 [11–17]
Gender % (F/M)	72% (13/18)
Employment	
Employed within last year	83% (15/18)
Months employed within last year	7.44 ± 4.8 [0–12]
Clinical characteristics	
Standard drinks per drinking day	8.41 ± 6.02 [2.71 – 24]
Years since alcohol-related problems began	8.63 ± 9.97 [1–38]
ADS score	15.44 ± 7.81 [9–42]
PACs craving score	11.53 ± 5.50 [4–24]
ISI	8 ± 6.22 [1–19]
DASS-21: Anxiety	3.28 ± 2.72 [0–9]
DASS-21: Depression	5.39 ± 4.98 [0–16]
DASS-21: Stress	7.06 ± 4.87 [0–17]
Comorbidities (Mini) %	33% (6/18)
Antidepressants (last 28 days) %	33% (6/18)
Tobacco use (last 28 days) %	22% (4/18)

Mean ± *SD*s [Range] format used to represent data unless otherwise noted

*ADS* alcohol dependence score, *PACS* penns alcohol craving score, *ISI* insomnia severity index, *DASS* depression anxiety symptom scale

**Fig. 2:**
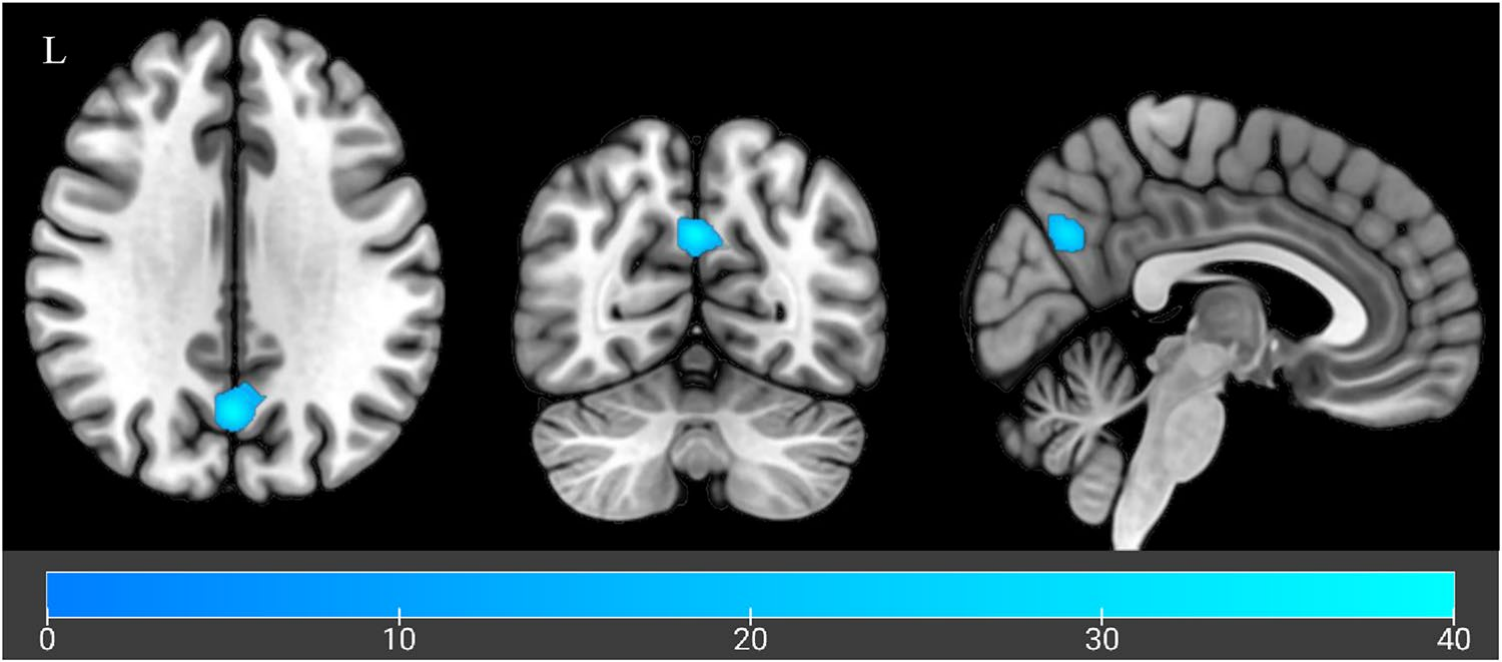
Whole brain analysis visualisation. Cluster hypoactivation across Alcohol and Neutral cues when comparing CBD to Placebo. Colour bar indicates F-values with cooler colours indicating increasing hypoactivation

### Cognitive functioning

For the BART (p = 0. 4) and CCT (p = 0. 57), there were no significant effects of CBD vs placebo. For the TMT, neither part-A, part-B or B-A times were significantly different between CBD and Pla dosing sessions (p > 0.62).

### Mood and subjective craving

There were no statistically significant CBD vs placebo treatment differences on the PANAS either before the scan (negative p = 0.170; positive p = 0.725) or after the scan (negative p = 0.69; positive p = 0.96). Overall, there was a significant increase between pre and post scan PANAS score for positive subscale (p < 0.001), but not negative subscale (p = 0.500), independent of treatment (negative p = 0.804; positive p = 0. 503).

There were no statistically significant differences in AUQ scores pre and post scan (p = 0.058) nor was this mediated by drug. No significant (CBD vs placebo) differences were observed pre or post scan timepoints (p = 0.40 and p = 0.49 respectively) nor was the difference between pre and post scan moderated by drug (p = 0.88). There was a significant increase in VAS scores following alcohol blocks vs control blocks (p < 0.001) but no interaction between treatment and block effect suggesting no differences in-scanner subjective craving (Alc vs Con p = 0.728) (Table 2).

**Table 2:** Cue Reactivity relevant AUQ and VAS scores

Craving measures		Placebo	CBD
AUQ	Pre-scan	1.54 ± 0.76	1.56 ± 0.99
	Post-scan	1.73 ± 1.13	1.75 ± 1.03
VAS craving	Alcohol images	2.44 ± 2.15	3.19 ± 2.84
	Control images	1.68 ± 2.18	± 2.53

Mean ± *SD*s format used to represent data unless otherwise noted

*AUQ* alcohol urge questionnaire, *VAS* visual analogue scale

### fMRI cue reactivity

#### ROI analysis

Compared to placebo, CBD non-significantly modulated activation in any a priori selected ROIs compared to placebo during alcohol cue (p_FWE_ > 0.043) or control cue presentations (p_FWE_ > 0.13). GEE results evaluating treatment effects on activation within a priori selected ROIs (left and right dorsolateral PFC, left and right caudate, and bilateral ventromedial) are presented in Supplementary Materials.

#### Whole brain analysis

There were clusters demonstrating overall alcohol cue reactivity for the sample, with increased activation to alcohol versus control cues, with one cluster (MNI peak coordinates − 5, − 91, − 9, p_FWE_ = 0.03) spanning the left and right lingual gyrus as well as the cuneus and a second left superior parietal lobule cluster (MNI peak coordinates − 35, − 61, 56, p_FWE_ = 0.042) see Table 3.

**Table 3:** Whole brain identified brain regions with significant differences

Side	Area	cluster size	MNI coordinates (max peak)			P_FWEc_
			X	Y	Z	
Condition						
Left/Right	Lingual Gyrus, Cuneus,	329	− 5	− 91	− 9	0.03
Left	Superior Parietal Lobule	276	− 35	− 61	56	0.042
Treatment						
Left	Precuneus	191	− 1	− 67	32	0.038

Clusters with statistically significant treatment differences in BOLD signal across Alcohol and Neutral cues compared to fixation

When evaluating treatment effects (CBD vs placebo), CBD sessions were associated with significant hypoactivation for sessions in a cluster within the precuneus (MNI peak coordinates: − 1, − 67, 32, p_FWE_ = 0.038; Fig. 2). This hypoactivation was evident across both control and alcohol cues, suggesting a non cue specific reduction within this cluster following CBD administration.

## Discussion

This is the first pharmaco-fMRI crossover design evaluating the neural effects of CBD on alcohol cue reactivity in non-treatment seeking participants with AUD. While there were no treatment effects seen for fMRI cue reactivity in the a priori defined regions (chiefly mesocorticolimbic) implicated in alcohol cue reactivity, whole brain analyses demonstrated CBD mediated reductions in cue reactivity relative to placebo, in a cluster of the precuneus during alcohol as well as control cue exposure blocks.

Non-cue-specific reduced activation observed following CBD administration suggests a general attenuation of brain activation in response to external cues. Previous studies have also observed that CBD modulated activation in the precuenous, but in tasks relating to verbal learning [66]. The precuneus is located between the sensorimotor cortices of the para-central lobule and the parieto-occipital cortex and is linked with subcortical and cortical structures. It is implicated as a hub serving the default mode network and the para-cingulate network, a subnetwork of the central executive network [67]. The precuneus is involved in episodic memory retrieval, visuospatial processing, and self-mental imagery [68], which are key processes in alcohol cue reactivity [69]. The precuneus has also been implicated in higher-order aspects of craving, including the subjective experience of craving [69]. Futhermore, previous cue reactivity studies have identified a relationship between severity of dependence and activation of the precuneus [69]. Therefore, given we did not observe any effects of CBD on subjective craving measures, it is plausible that greater treatment differences may emerge in treatment seeking samples and among participants with greater dependence severity.

We found no evidence of performance differences between CBD and placebo across neuropsychological tests, including the BART, CCT, TMT-B or TMT-B-A tasks. These results align with previous literature demonstrating a lack of CBD-mediated modulation of executive functioning in individuals with opioid dependence (as measured using the Digit Symbol Substitution Task, Digit Span Test–Backward, and a Continuous Performance Task) [24] or during tobacco abstinence [70]. Taken together, these results suggest that CBD has minimal impact on executive functioning in individuals with substance use disorders. This is relevant to note given that cognitive deficits are common among treatment-seeking individuals [71] and can hinder medication compliance or response to psychological treatment [72].

The present study has both strengths and limitations. It is the first study, to our knowledge, to examine the effects of CBD on individuals with AUD with a robust crossover design, which reduces within-participant variability. Our study design included a substantial washout period and used GEE to ensure no carry-over effects of CBD. Additionally, we employed multi-echoplanar imaging which performs better [73–75] and recovers more orbito-frontal signal [76] compared to single band acquisitions. However, limitations include the potentially low generalizability of results due to the recruitment of non-treatment-seeking participants with less severe clinical presentations. The lack of statistically significant CBD-modulated changes in ROIs and clusters in our whole brain analysis may be attributed to reduced treatment effects observed in non-treatment seekers [77, 78]. Nonetheless, the current sample exhibited a baseline consumption of eight standard drinks per drinking day, which, although is substantially less than the AUD treatment-seeking population in Australian clinical trials [79, 80], exceeds the national alcohol consumption guidelines [81] and falls within the high risk category of World Health Organisation risk levels (82).

In conclusion, in non treatment seeking individuals with AUD, high dose CBD (800 mg/day) modulates precuneus activation in response to non-specific cues relative to placebo. CBD did not significantly change subjective craving relative to placebo and did not impair executive function or change self-reports of positive or negative affect. Further investigation in AUD treatment-seeking populations are warranted to determine the potential of CBD for the management of AUD.

## Supplementary Information

Below is the link to the electronic supplementary material.

Supplementary file1 (DOCX 241 KB)

## Data Availability

Data sets generated during the current study are available on request from the corresponding author.
