# Supplementary material for: Cannabidiol attenuates precuneus activation during appetitive cue exposure in individuals with alcohol use disorder
Source: Eur Arch Psychiatry Clin Neurosci. 2025 Mar 18;275(7):2129–39. doi: 10.1007/s00406-025-01983-4 (PMC12589260; doi:10.1007/s00406-025-01983-4)
Supplement: Supplementary file 1 — Supplementary file1 (DOCX 241 KB) [file 406_2025_1983_MOESM1_ESM.docx]

Cue reactivity imaging task layout.


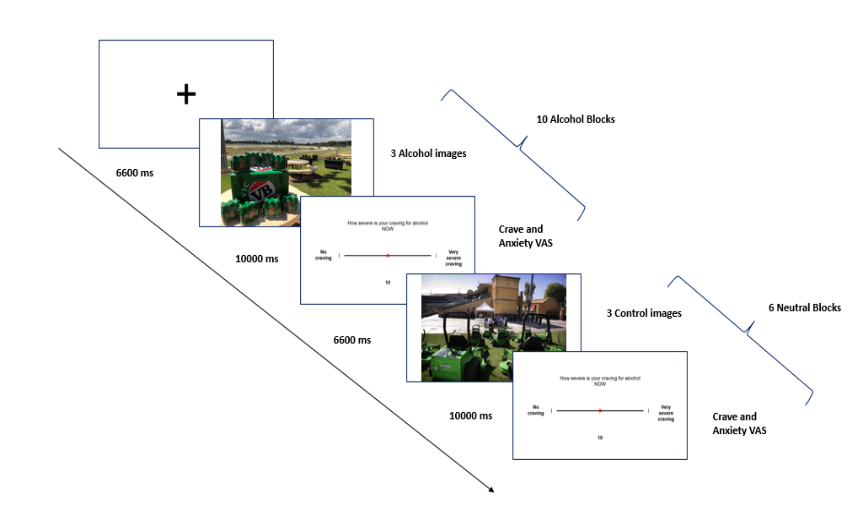


Imaging data preprocessing

fMRIPrep boiler plate

A total of 2 T1-weighted (T1w) images were found within the input BIDS dataset. All of them were corrected for intensity non-uniformity (INU) with N4BiasFieldCorrection (Tustison et al. 2010), distributed with ANTs 2.3.3 (Avants et al. 2008, RRID:SCR_004757). The T1w-reference was then skull-stripped with a Nipype implementation of the antsBrainExtraction.sh workflow (from ANTs), using OASIS30ANTs as target template. Brain tissue segmentation of cerebrospinal fluid (CSF), white-matter (WM) and gray-matter (GM) was performed on the brain-extracted T1w using fast (FSL 5.0.9, RRID:SCR_002823, Zhang, Brady, and Smith 2001). A T1w-reference map was computed after registration of 2 T1w images (after INU-correction) using mri_robust_template (FreeSurfer 6.0.1, Reuter, Rosas, and Fischl 2010). Brain surfaces were reconstructed using recon-all (FreeSurfer 6.0.1, RRID:SCR_001847, Dale, Fischl, and Sereno 1999), and the brain mask estimated previously was refined with a custom variation of the method to reconcile ANTs-derived and FreeSurfer-derived segmentations of the cortical gray-matter of Mindboggle (RRID:SCR_002438, Klein et al. 2017). Volume-based spatial normalization to one standard space (MNI152NLin2009cAsym) was performed through nonlinear registration with antsRegistration (ANTs 2.3.3), using brain-extracted versions of both T1w reference and the T1w template. The following template was selected for spatial normalization: ICBM 152 Nonlinear Asymmetrical template version 2009c [Fonov et al. (2009), RRID:SCR_008796; TemplateFlow ID: MNI152NLin2009cAsym],

*Functional data preprocessing*

For each of the 2 BOLD runs found per subject (across all tasks and sessions), the following preprocessing was performed. First, a reference volume and its skull-stripped version were generated from the shortest echo of the BOLD run using a custom methodology of fMRIPrep. Head-motion parameters with respect to the BOLD reference (transformation matrices, and six corresponding rotation and translation parameters) are estimated before any spatiotemporal filtering using mcflirt (FSL 5.0.9, Jenkinson et al. 2002). A B0-nonuniformity map (or fieldmap) was directly measured with an MRI scheme designed with that purpose (typically, a spiral pulse sequence). The fieldmap was then co-registered to the target EPI (echo-planar imaging) reference run and converted to a displacements field map (amenable to registration tools such as ANTs) with FSL’s fugue and other SDCflows tools. Based on the estimated susceptibility distortion, a corrected EPI (echo-planar imaging) reference was calculated for a more accurate co-registration with the anatomical reference. The BOLD reference was then co-registered to the T1w reference using bbregister (FreeSurfer) which implements boundary-based registration (Greve and Fischl 2009). Co-registration was configured with six degrees of freedom. The BOLD time-series (including slice-timing correction when applied) were resampled onto their original, native space by applying a single, composite transform to correct for head-motion and susceptibility distortions. These resampled BOLD time-series will be referred to as preprocessed BOLD in original space, or just preprocessed BOLD. A T2* map was estimated from the preprocessed BOLD by fitting to a monoexponential signal decay model with nonlinear regression, using T2*/S0 estimates from a log-linear regression fit as initial values. For each voxel, the maximal number of echoes with reliable signal in that voxel were used to fit the model. The calculated T2* map was then used to optimally combine preprocessed BOLD across echoes following the method described in (Posse et al. 1999). The optimally combined time series was carried forward as the preprocessed BOLD. First, a reference volume and its skull-stripped version were generated using a custom methodology of fMRIPrep. The BOLD time-series were resampled into standard space, generating a preprocessed BOLD run in MNI152NLin2009cAsym space. First, a reference volume and its skull-stripped version were generated using a custom methodology of fMRIPrep. Several confounding time-series were calculated based on the preprocessed BOLD: framewise displacement (FD), DVARS and three region-wise global signals. FD was computed using two formulations following Power (absolute sum of relative motions, Power et al. (2014)) and Jenkinson (relative root mean square displacement between affines, Jenkinson et al. (2002)). FD and DVARS are calculated for each functional run, both using their implementations in Nipype (following the definitions by Power et al. 2014). The three global signals are extracted within the CSF, the WM, and the whole-brain masks. Additionally, a set of physiological regressors were extracted to allow for component-based noise correction (CompCor, Behzadi et al. 2007). Principal components are estimated after high-pass filtering the preprocessed BOLD time-series (using a discrete cosine filter with 128s cut-off) for the two CompCor variants: temporal (tCompCor) and anatomical (aCompCor). tCompCor components are then calculated from the top 2% variable voxels within the brain mask. For aCompCor, three probabilistic masks (CSF, WM and combined CSF+WM) are generated in anatomical space. The implementation differs from that of Behzadi et al. in that instead of eroding the masks by 2 pixels on BOLD space, the aCompCor masks are subtracted a mask of pixels that likely contain a volume fraction of GM. This mask is obtained by dilating a GM mask extracted from the FreeSurfer’s aseg segmentation, and it ensures components are not extracted from voxels containing a minimal fraction of GM. Finally, these masks are resampled into BOLD space and binarized by thresholding at 0.99 (as in the original implementation). Components are also calculated separately within the WM and CSF masks. For each CompCor decomposition, the k components with the largest singular values are retained, such that the retained components’ time series are sufficient to explain 50 percent of variance across the nuisance mask (CSF, WM, combined, or temporal). The remaining components are dropped from consideration. The head-motion estimates calculated in the correction step were also placed within the corresponding confounds file. The confound time series derived from head motion estimates and global signals were expanded with the inclusion of temporal derivatives and quadratic terms for each (Satterthwaite et al. 2013). Frames that exceeded a threshold of 0.5 mm FD or 1.5 standardised DVARS were annotated as motion outliers. All resamplings can be performed with a single interpolation step by composing all the pertinent transformations (i.e. head-motion transform matrices, susceptibility distortion correction when available, and co-registrations to anatomical and output spaces). Gridded (volumetric) resamplings were performed using antsApplyTransforms (ANTs), configured with Lanczos interpolation to minimize the smoothing effects of other kernels (Lanczos 1964). Non-gridded (surface) resamplings were performed using mri_vol2surf (FreeSurfer).

Many internal operations of fMRIPrep use Nilearn 0.6.2 (Abraham et al. 2014, RRID:SCR_001362), mostly within the functional processing workflow. For more details of the pipeline, see [the section corresponding to workflows in fMRIPrep’s documentation](https://fmriprep.readthedocs.io/en/latest/workflows.html).

Procedure Flow chart

> 18 day washout

Placebo (n = 10)

CBD (n = 9)

Medical screen, Laboratory evaluations

Randomisation

Medical review: Review side effects

Phone Screening: Exclusion criteria; Inclusion criteria; Mini Neuropsychiatric

Session 2: Repeat T1, T2, T3

T4: Follow up

**Executive Functioning Tasks**

**Pre scan:** Craving and Mood

**Scan:** Structural Scan; B0 map; Functional Cue Reactivity

**Post scan**: Craving and Mood

Pre-screen/Screen/Randomisation (Day 0)

Read PIS and sign consent form

Sample characteristics questionnaires (e.g. demographics questions, drinking history)

**1.30 (75min)**

**2.45 (15 min)**

**Baseline:** Craving and Mood

T2: Brain Imaging (Day 2)

**0.15 (60min)**

T1: Questionnaires (Day 1)

**1.15 (15 min)**

**0.00 (15 min)**

**Executive Functioning Tasks**

**Psychophysiological Cue Reactivity**

**Debrief**

**1.30 (60 min)**

**2.30 (15 min)**

**Baseline:** Craving and Mood

**0.15 (75 min)**

**0.00 (15min)**

CBD (n = 11)

Placebo (n = 11)

T3: Psychophysiology (Day 3)

> 18 day washout

*Note. Data collected from T3 not presented in this paper.*

Generalized Estimating Equations (GEE) Results for ROI Activation During Alcohol Cue fMRI Task Blocks

|  | BL VMPFC | | L caudate | | R caudate | | L DLPFC | | R DLPFC | |
| --- | --- | --- | --- | --- | --- | --- | --- | --- | --- | --- |
|  | estimate (SD) | Pr(>\|W\|) | estimate (SD) | Pr(>\|W\|) | estimate (SD) | Pr(>\|W\|) | estimate (SD) | Pr(>\|W\|) | estimate (SD) | Pr(>\|W\|) |
| (Intercept) | 0.1328 (0.1443) | 0.3575 | -0.2006 (0.2031) | 0.32 | -0.1174 (0.1444) | 0.42 | -0.0417 (0.1544) | 0.787 | -0.0350 (0.2557) | 0.89 |
| Session 1 | 0.1898 (0.1986) | 0.339 | -0.0094 (0.1307) | 0.94 | 0.0392 (0.1356) | 0.77 | -0.4353 (0.1976) | **0.028** | -0.3979 (0.3212) | 0.22 |
| Carry 2 | -0.7117 (0.3673) | 0.0526 | -0.2432 (0.3509) | 0.49 | -0.3503 (0.3554) | 0.32 | 0.0508 (0.2922) | 0.862 | -0.5509 (0.5391) | 0.31 |
| Drug 1 | -0.4597 (0.2275) | **0.0433** | -0.0832 (0.2225) | 0.71 | -0.2153 (0.1799) | 0.23 | 0.0220 (0.2279) | 0.923 | -0.2282 (0.3726) | 0.54 |
| DDB | 0.0257 (0.1450) | 0.8592 | 0.1309 (0.1736) | 0.45 | 0.0224 (0.1498) | 0.88 | 0.1115 (0.1460) | 0.445 | 0.0639 (0.2922) | 0.83 |

Note. Results from the Generalized Estimating Equations (GEE) model evaluating the effects of treatment, session order, carryover effects, and drinking history on regions of interest (ROI) activation during alcohol cue fMRI task blocks. ROIs included the bilateral ventromedial prefrontal cortex (BL VMPFC), left (L) and right (R) caudate, and left (L) and right (R) dorsolateral prefrontal cortex (DLPFC). The model included the following variables:

- Session 1: Session order effect.
- Carry 2: Carryover effect (AB/BA).
- Drug 1: Treatment with cannabidiol (CBD).
- DDB: Drinks consumed in the previous 24 hours.

The GEE model was specified as follows:

ROI ∼ Session Order + Carryover Effect + Treatment (CBD) + Drinks in Previous 24 Hours

An exchangeable working correlation structure was used to estimate treatment effects, controlling for session order and carryover effects.

Generalized Estimating Equations (GEE) Results for ROI Activation During Control Cue fMRI Task Blocks

|  | BL VMPFC | | L caudate | | R caudate | | L DLPFC | | R DLPFC | |
| --- | --- | --- | --- | --- | --- | --- | --- | --- | --- | --- |
|  | estimate (SD) | Pr(>\|W\|) | estimate (SD) | Pr(>\|W\|) | estimate (SD) | Pr(>\|W\|) | estimate (SD) | Pr(>\|W\|) | estimate (SD) | Pr(>\|W\|) |
| Intercept | -0.0767 (0.1633) | 0.64 | -0.2509 (0.2167) | 0.25 | -0.1772 (0.1484) | 0.23 | -0.0972 (0.1518) | 0.52 | -0.0621 (0.2452) | 0.8 |
| Session 1 | 0.1906 (0.1377) | 0.17 | 0.0041 (0.1128) | 0.97 | 0.0432 (0.1148) | 0.71 | -0.3846 (0.1958) | 0.05 | -0.3518 (0.3595) | 0.33 |
| Carry 2 | -0.5161 (0.3169) | 0.1 | -0.2068 (0.3423) | 0.55 | -0.2968 (0.3287) | 0.37 | 0.0555 (0.2748) | 0.84 | -0.5645 (0.5475) | 0.3 |
| Drug 1 | -0.2930 (0.1924) | 0.13 | -0.0476 (0.2254) | 0.83 | -0.1667 (0.1667) | 0.32 | 0.0268 (0.2208) | 0.9 | -0.2298 (0.3972) | 0.56 |
| DDB | 0.1308 (0.1319) | 0.32 | 0.1342 (0.1738) | 0.44 | 0.0407 (0.1438) | 0.78 | 0.1113 (0.1408) | 0.43 | 0.0204 (0.2785) | 0.94 |

Note. Results from the Generalized Estimating Equations (GEE) model evaluating the effects of treatment, session order, carryover effects, and drinking history on regions of interest (ROI) activation during control cue fMRI task blocks. ROIs included the bilateral ventromedial prefrontal cortex (BL VMPFC), left (L) and right (R) caudate, and left (L) and right (R) dorsolateral prefrontal cortex (DLPFC). The model included the following variables:

- Session 1: Session order effect.
- Carry 2: Carryover effect (AB/BA).
- Drug 1: Treatment with cannabidiol (CBD).
- DDB: Drinks consumed in the previous 24 hours.

The GEE model was specified as follows:

ROI ∼ Session Order + Carryover Effect + Treatment (CBD) + Drinks in Previous 24 Hours

An exchangeable working correlation structure was used to estimate treatment effects, controlling for session order and carryover effects.

*Estimated Marginal Means for Brain ROI Activity During Alcohol and Control Cue Exposure: Results from Generalized Estimating Equation Models*


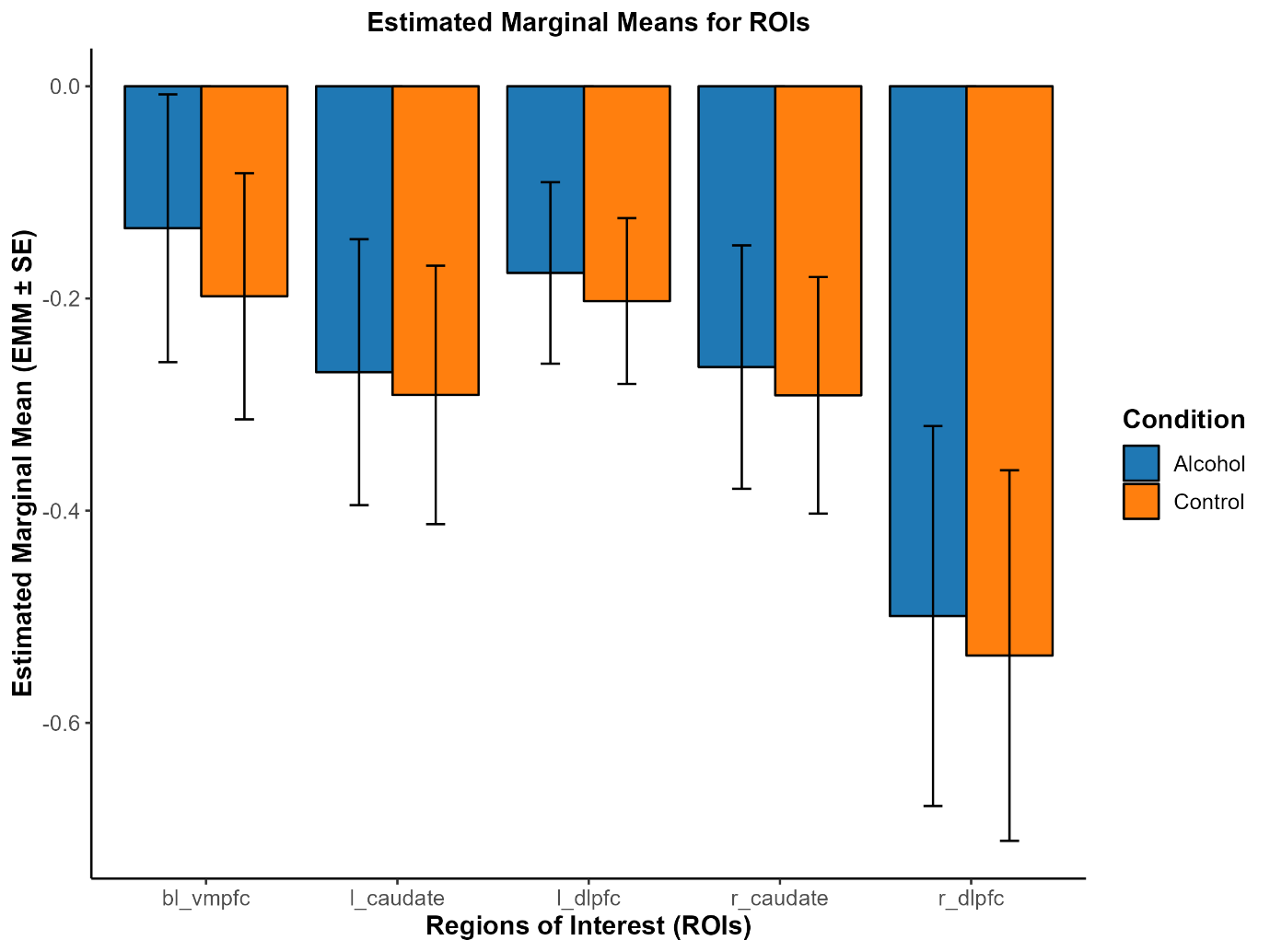


*Note*. Estimated marginal means extracted from generalised estimated equation models examining the elicited brain activity during alcohol and control cue exposure blocks during fmri cue reactivity task. ROIs included the bilateral ventromedial prefrontal cortex (BL VMPFC), left (L) and right (R) caudate, and left (L) and right (R) dorsolateral prefrontal cortex (DLPFC). The model included the following variables:

- Session order effect.
- Carryover effect (AB/BA).
- Drinks consumed in the previous 24 hours.

The GEE model was specified as follows:

*ROI ∼ Session Order + Carryover Effect + Drinks in Previous 24 Hours*
